# Supplementary material for: The H2A.Z histone variant integrates Wnt signaling in intestinal epithelial homeostasis
Source: Nat Commun. 2019 Apr 23;10:1827. doi: 10.1038/s41467-019-09899-z (PMC6478875; doi:10.1038/s41467-019-09899-z)
Supplement: Supplementary file 1 — Supplementary Information [file 41467_2019_9899_MOESM1_ESM.pdf]

Central role of H2A.Z in intestinal homeostasis : an epistatic relationship with CDX2 integrates Wnt signaling

Rispal J. *et al.*

Supplementary Information

siRNA serie 1

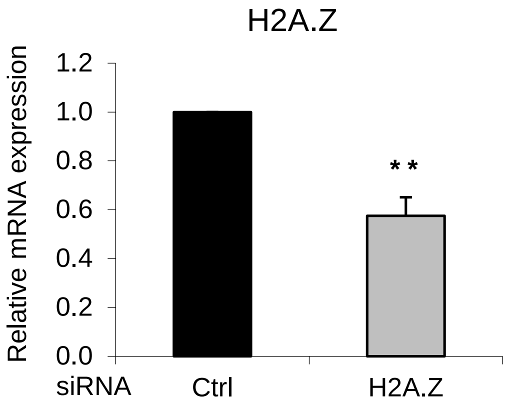

siRNA serie 2

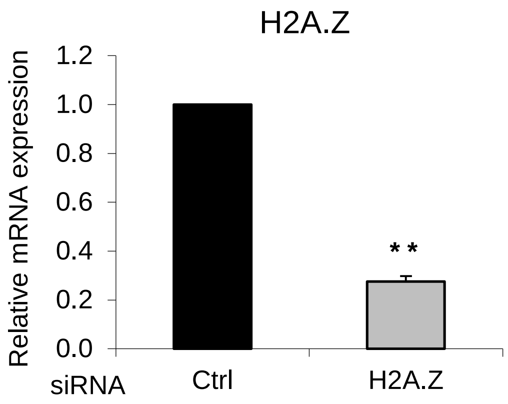

<u>Supplementary Figure 1</u> : Silencing, in HIEC cells, of siRNA-targeted H2A.Z messenger.
Two different series of siRNA were used to test the proliferation abilities of cells (see Figure 1A). The mean and standard error are shown (n=5 independent experiments). Statistical analysis was done using Student's t-Test (**, p<0.02 vs control siRNA).

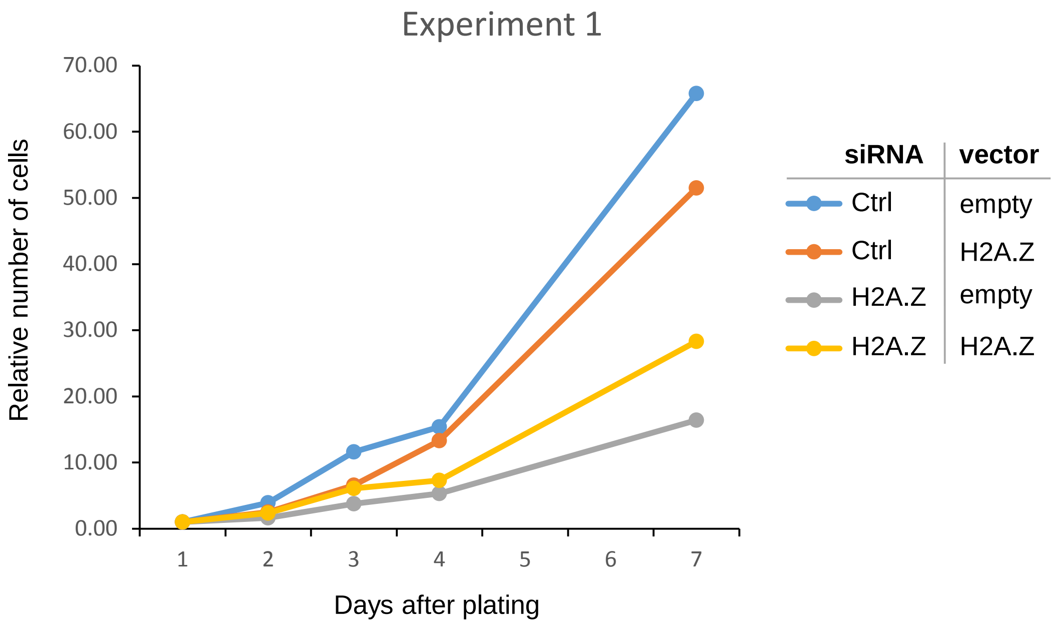

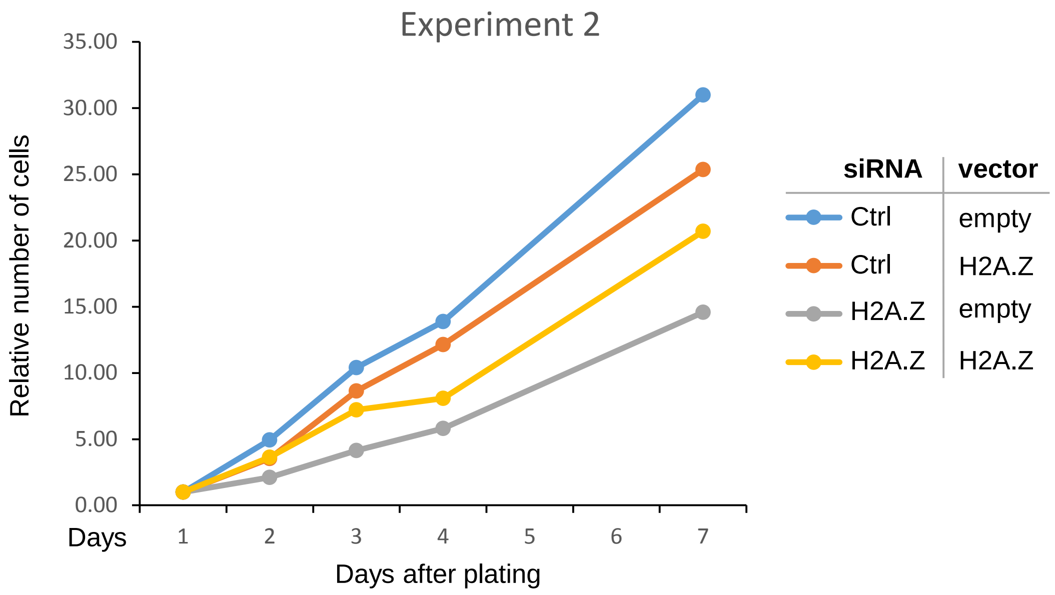

<u>Supplementary Figure 2</u> : Complementation of H2A.Z knock-down partially reverses the growth impairment. Caco-2/15 cells were transfected using siRNAs control or directed against H2A.Z and the cell number was measured and represented relative to 1 for the day 1 after transfection and plating. Two independent representative experiments are shown.

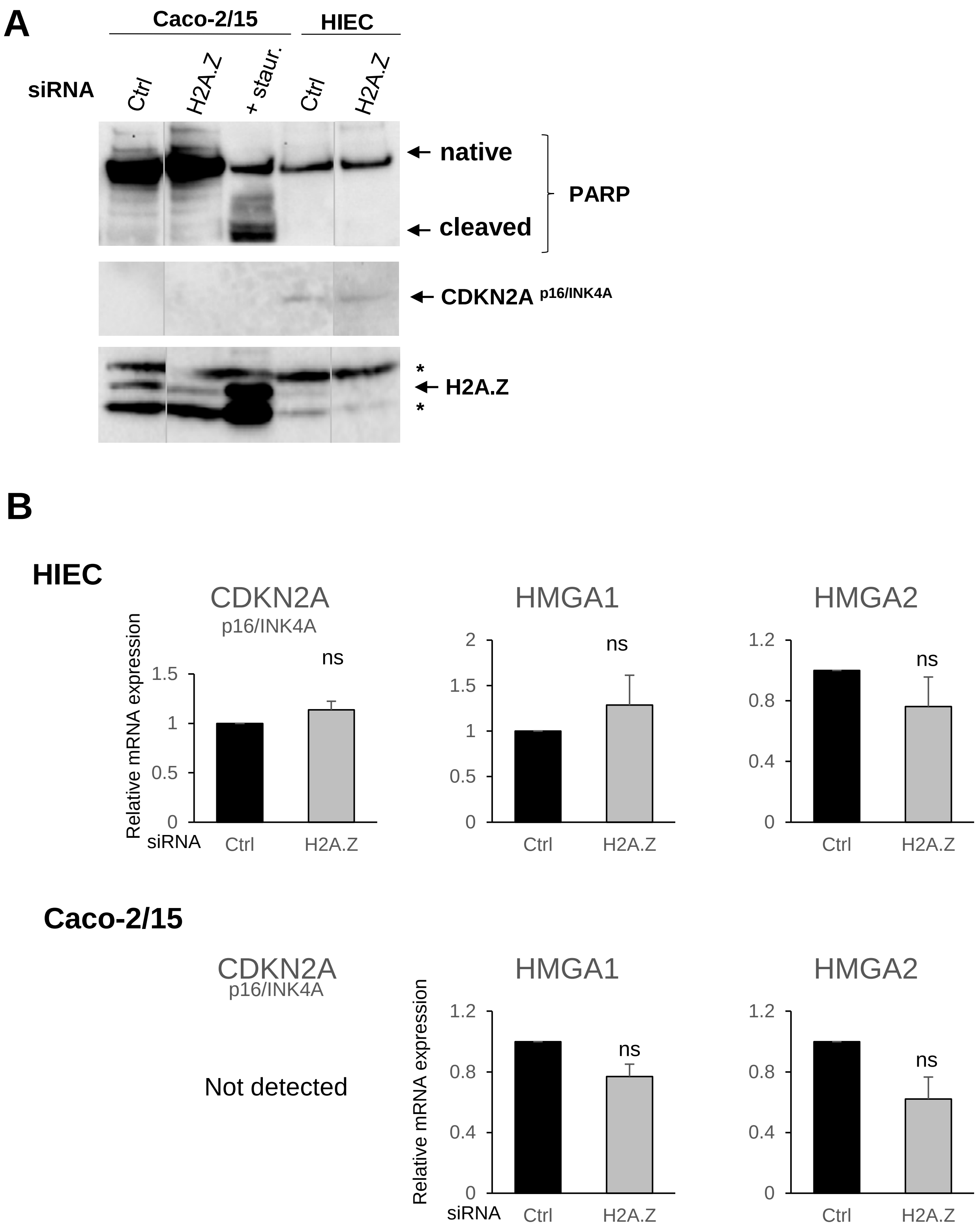

Supplementary Figure 3 : Expression of apoptosis and senescence markers upon siRNA treatment.
A) Caco-2/15 and HIEC cells were transfected using indicated siRNAs and proteins were harvested 3 days later and analyzed by western-blot using indicated antibodies. A batch of Caco-2/15 cells were treated using 200nM staurosporine (+staur) for 16 hours to induce apoptosis and serve as positive control. Stars indicate non specific bands. B) HIEC and Caco-2/15 cells were transfected using indicated siRNA. Three days later, mRNA were harvested and analyzed by RT-qPCR. The mean and standard error are shown (n=3 independent experiments).

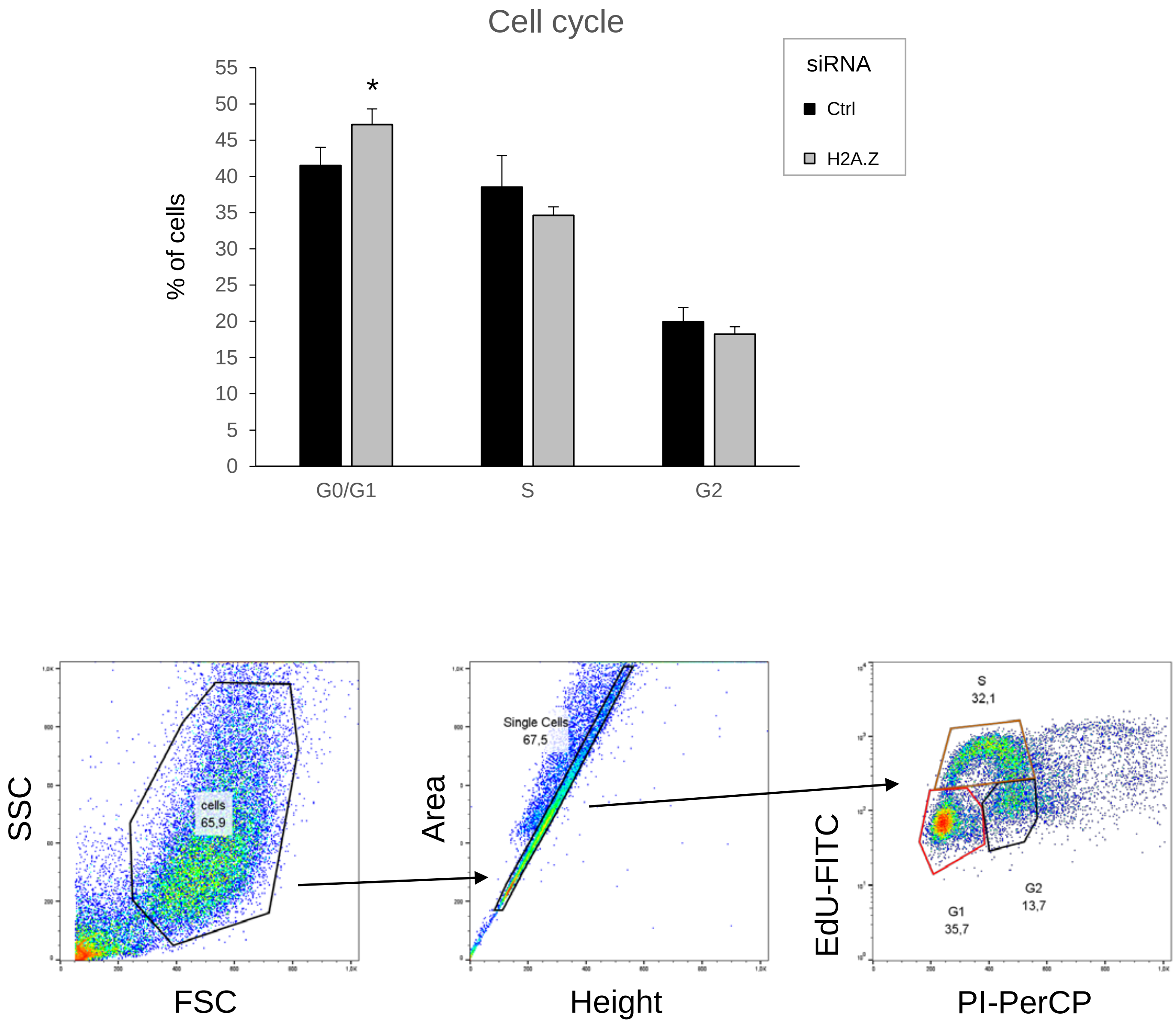

<u>Supplementary Figure 4</u> : Impact of H2A.Z knock-down on the cell cycle in Caco-2/15 cells.
Caco-2/15 cells were transfected using indicated siRNA. 72 hours later, cells were treated using EdU for 2 hours and harvested for cell cycle analysis (upper panel) by flow cytometry (using FACScalibur).
The mean and standard error from three independent experiment are shown (*, $p<0.05$ vs control siRNA).
The gating strategy is also shown in lower panels.

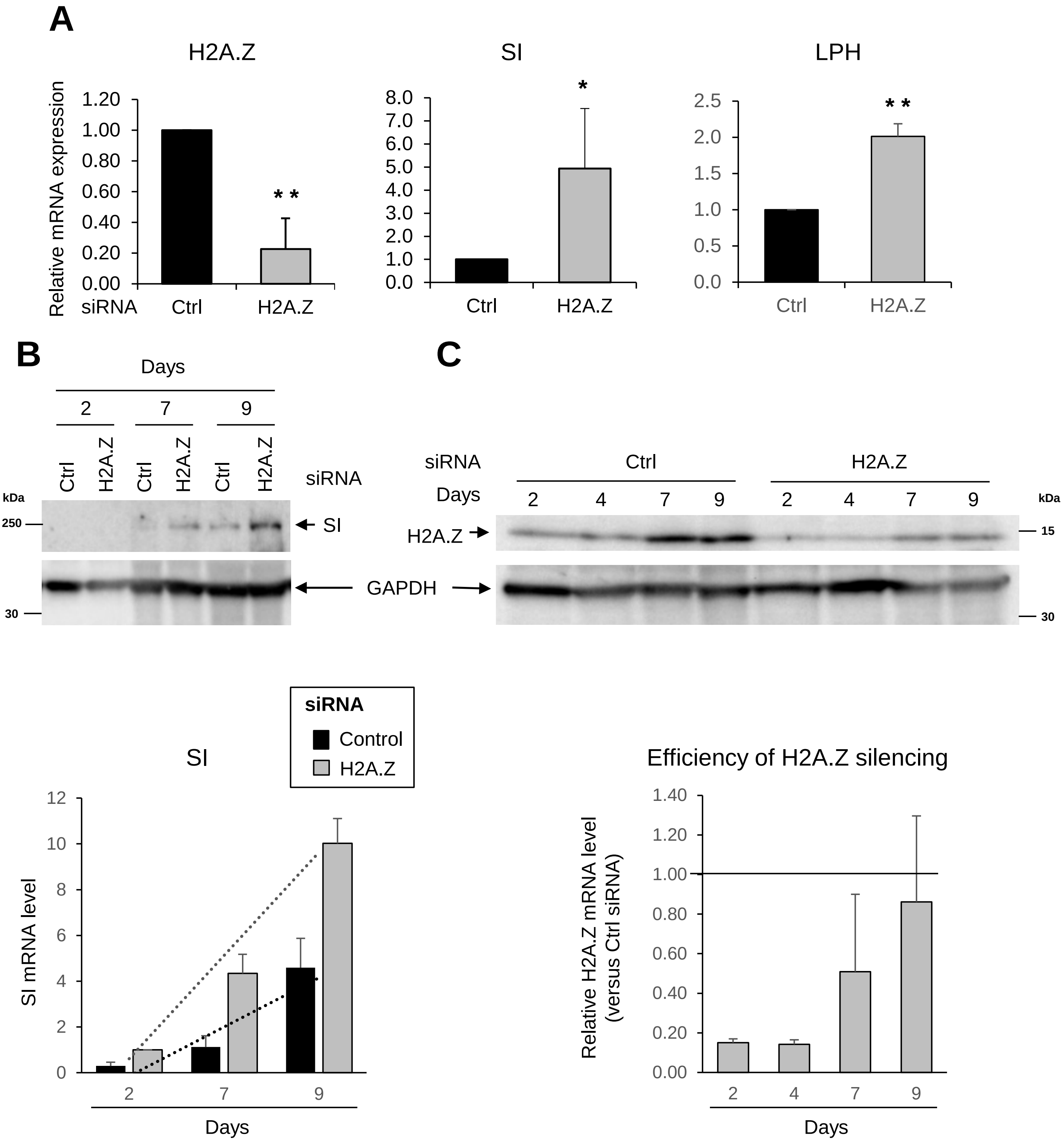

<u>**Supplementary Figure 5**</u> : Impact of H2A.Z knock-down on gene expression.
A) As in Figure 2A, gene expression in Caco-2/15 transfected using another H2A.Z-targetting siRNA (72h after transfection). B) Kinetic analysis of Sucrase-Isomaltase induction upon indicated siRNAs transfection, at mRNA (lower panel) or protein (upper one) levels. The mean of 3 independent experiments is shown for RT-qPCR experiments. C) As in B, kinetic analysis, by western blot (protein) and RT-qPCR (mRNA), of H2A.Z expression in Caco-2/15 transfected using H2A.Z siRNA at Day 0. Results for a representative experiment (western-blot) or the mean of 3 experiments (RT-qPCR) are shown.

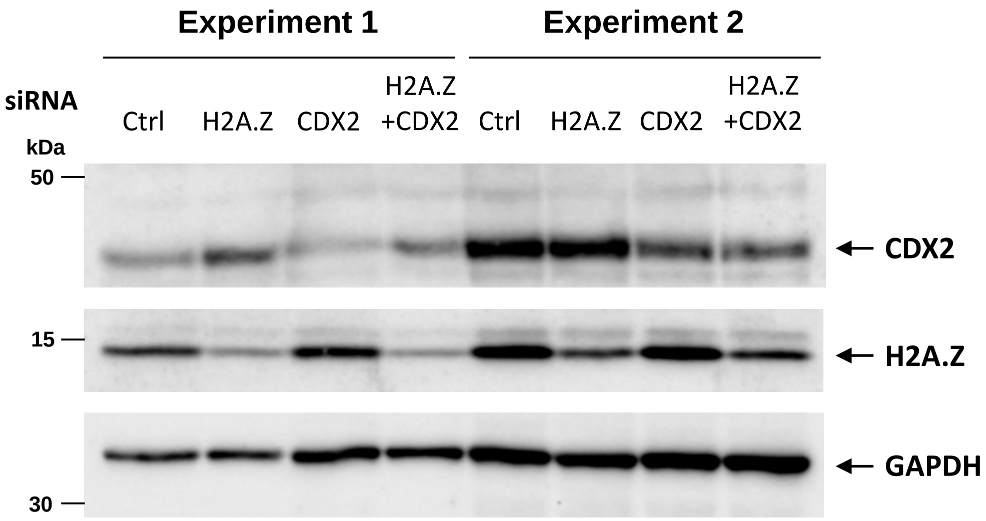

Supplementary Figure 6 : Western-blot analysis of H2A.Z and CDX2 protein levels upon siRNA treatments. Caco-2/15 cells were transfected using indicated siRNAs. Three days later, proteins were harvested and analyzed by western-blot using antiH2A.Z or anti-CDX2 antibodies. GAPDH serves as loading control. Results of two independent experiments are shown.

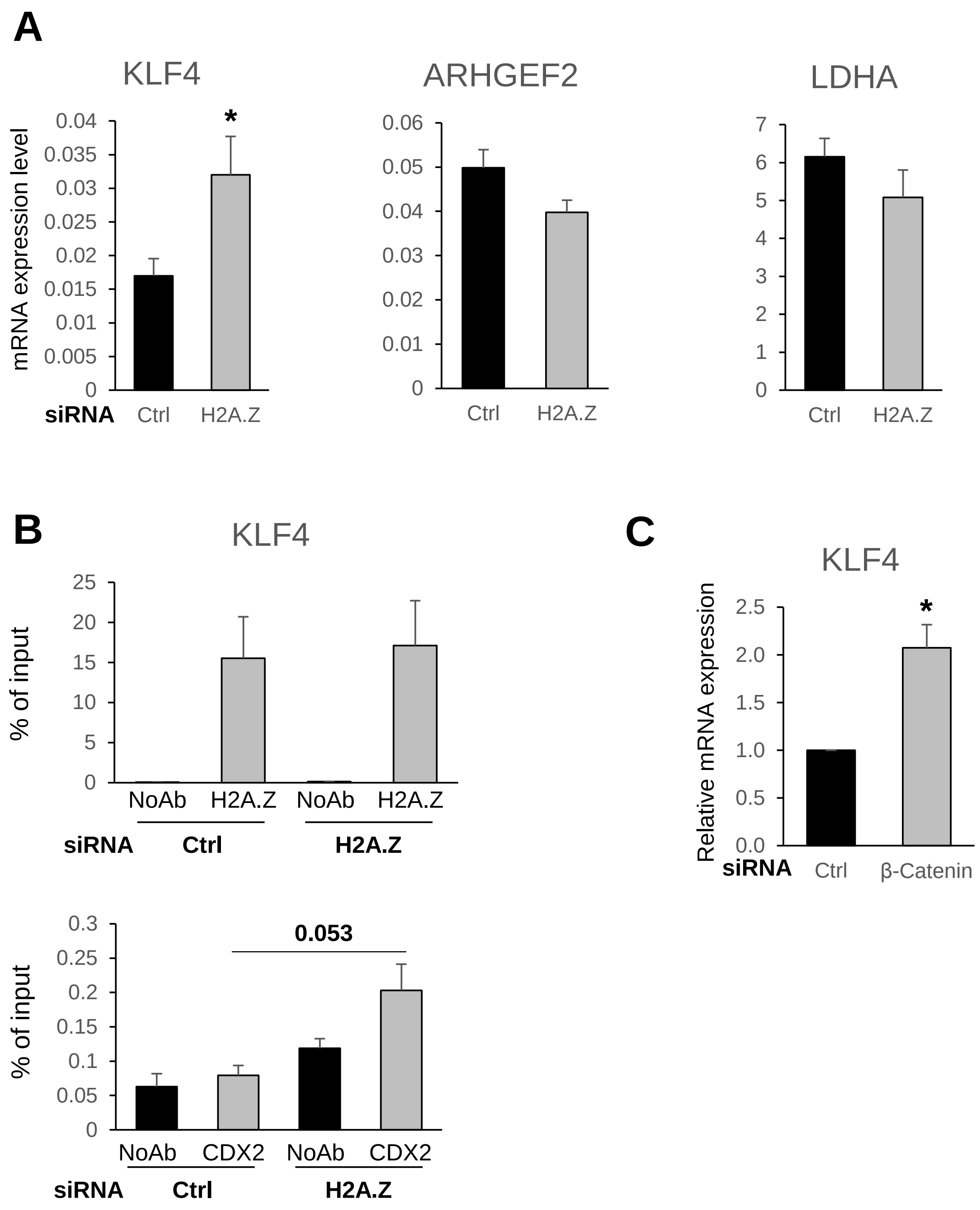

<u>Supplementary Figure 7</u> : Analysis of mRNA expression and promoter enrichment of other H2A.Z target genes.

A) mRNA of Caco-2/15 cells were obtained and analyzed by RT-qPCR for KLF4, ARHGEF2 and LDHA mRNA levels, as described for Figure 2A. B) As for Figures 5B and 5C, for promoter of *KLF4.* C) Same as Figure 6C for KLF4 expression.

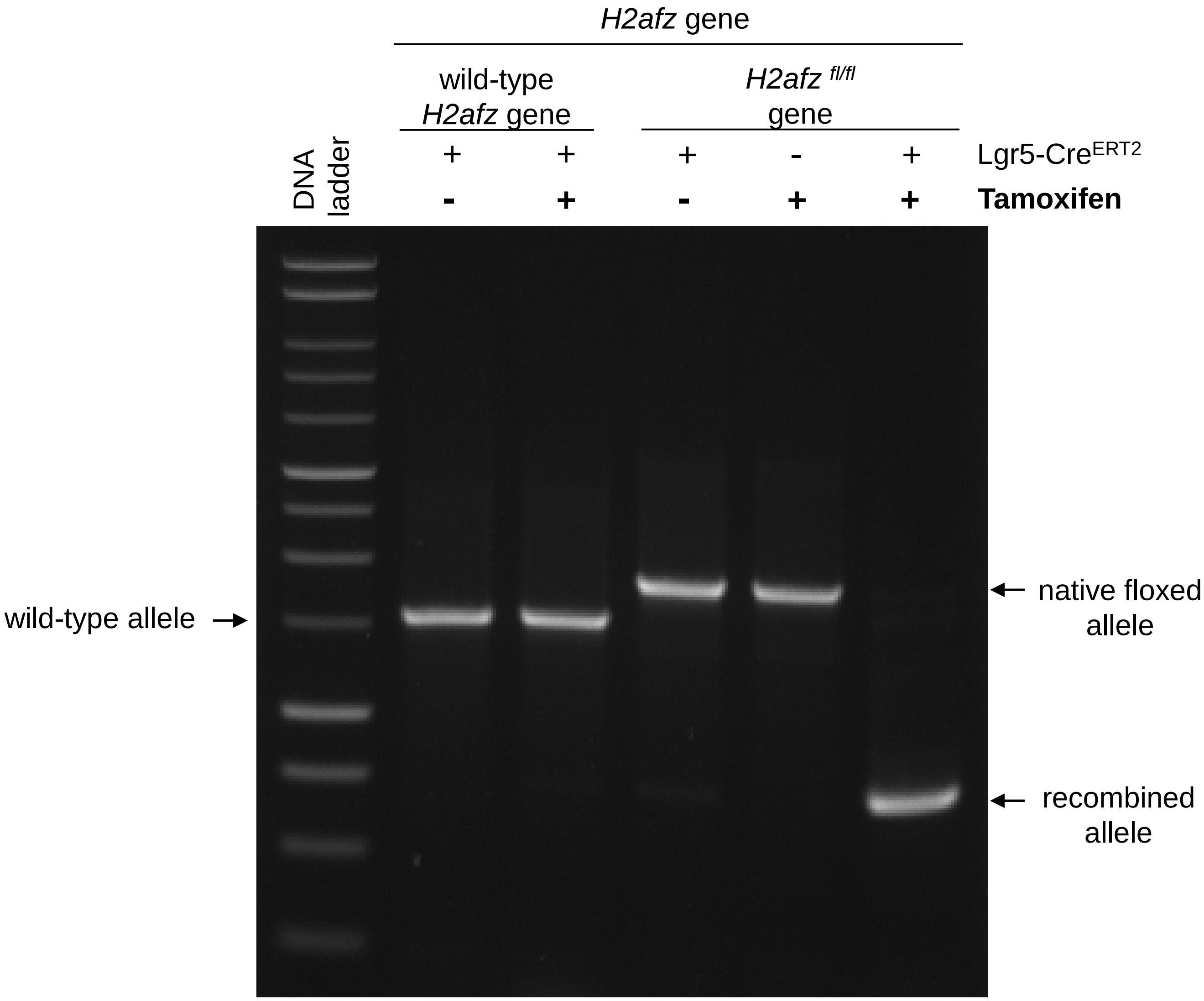

Supplementary Figure 8 : Genotyping of *H2afz* gene.

Genomic DNA extracted from intestinal epithelium of representative mice, habouring the indicated genotypes, and after Tamoxifen treatment or not, was nalyzed by PCR. The DNA recombination is observed only in *H2afz*$^{fl/fl}$ + *Lgr5-CRE*^{ERT2} strain upon Tamoxifen treatment.

Note however that the larger non-recombined allele will certainly have disadvantage for amplification compared to the shorter recombined band and could be minored in this panel (recombination efficiency may be overestimated).

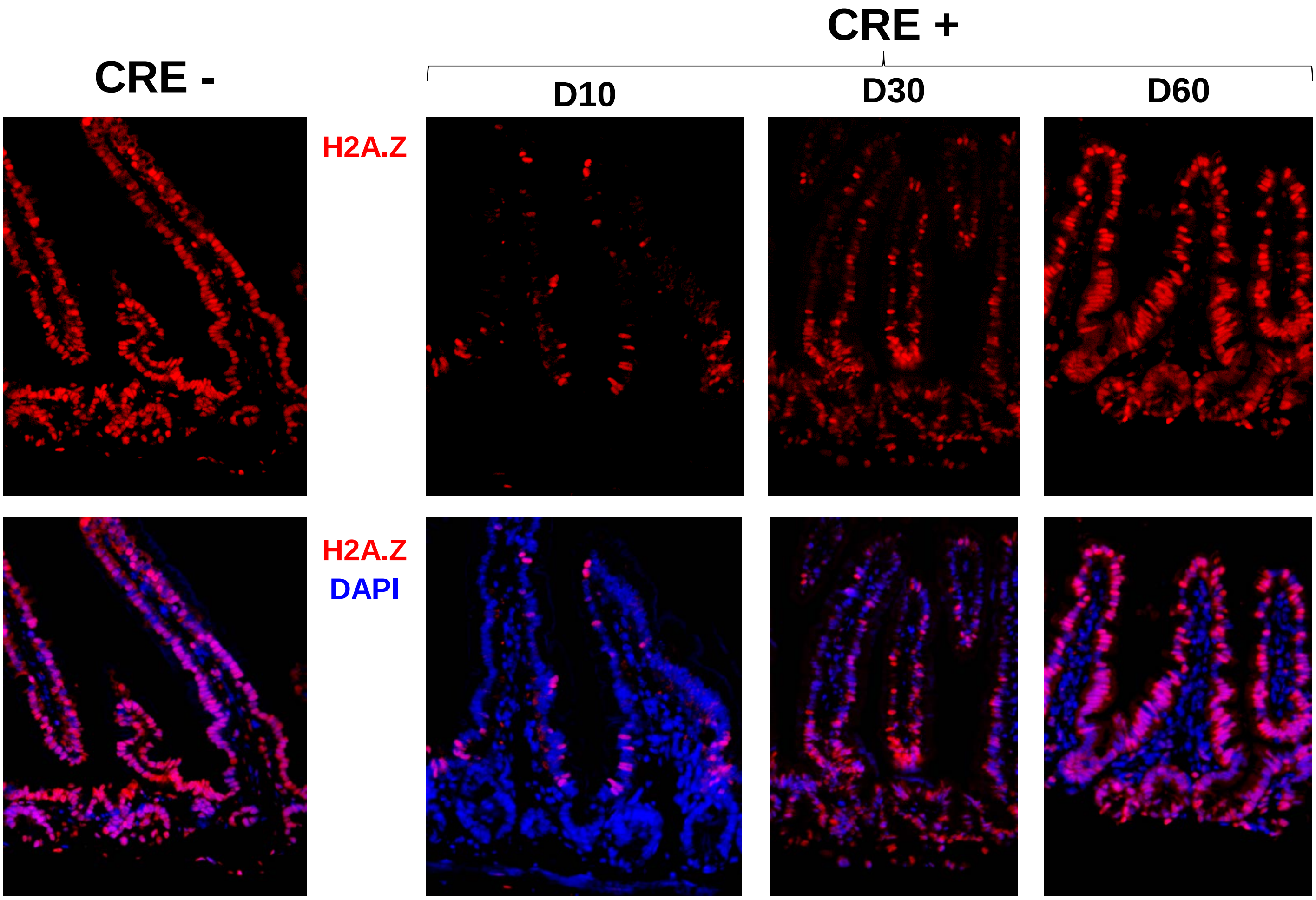

<u>Supplementary Figure 9</u> : Long-term evolution of H2A.Z staining in tamoxifen-induced mice.
Mice were subjected to tamoxifen treatment for 10 days, as described for Figure 3. Mice were then euthanized immediatley or after a 20 days or a 50 days recovery period (mice being feeded with normal food during these recovery periods). Then, intestine were dissected and stained using an H2A.Z antibody or DAPI. A representative panel for each time is shown.

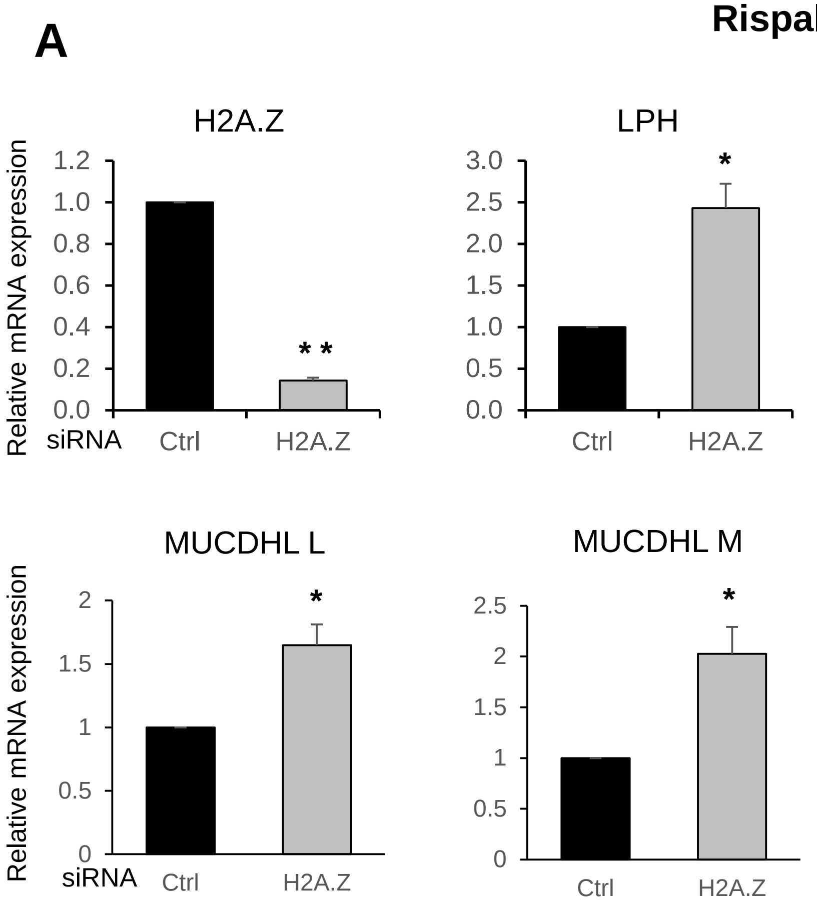

Supplementary Figure 10 : Gene expression upon siRNA-mediated H2A.Z silencing in Caco-2/15 cell samples used in ChIP experiments.

The mean and standard error of 3 experiments is shown (**, p<0.02; *, p<0.05 vs control siRNA).

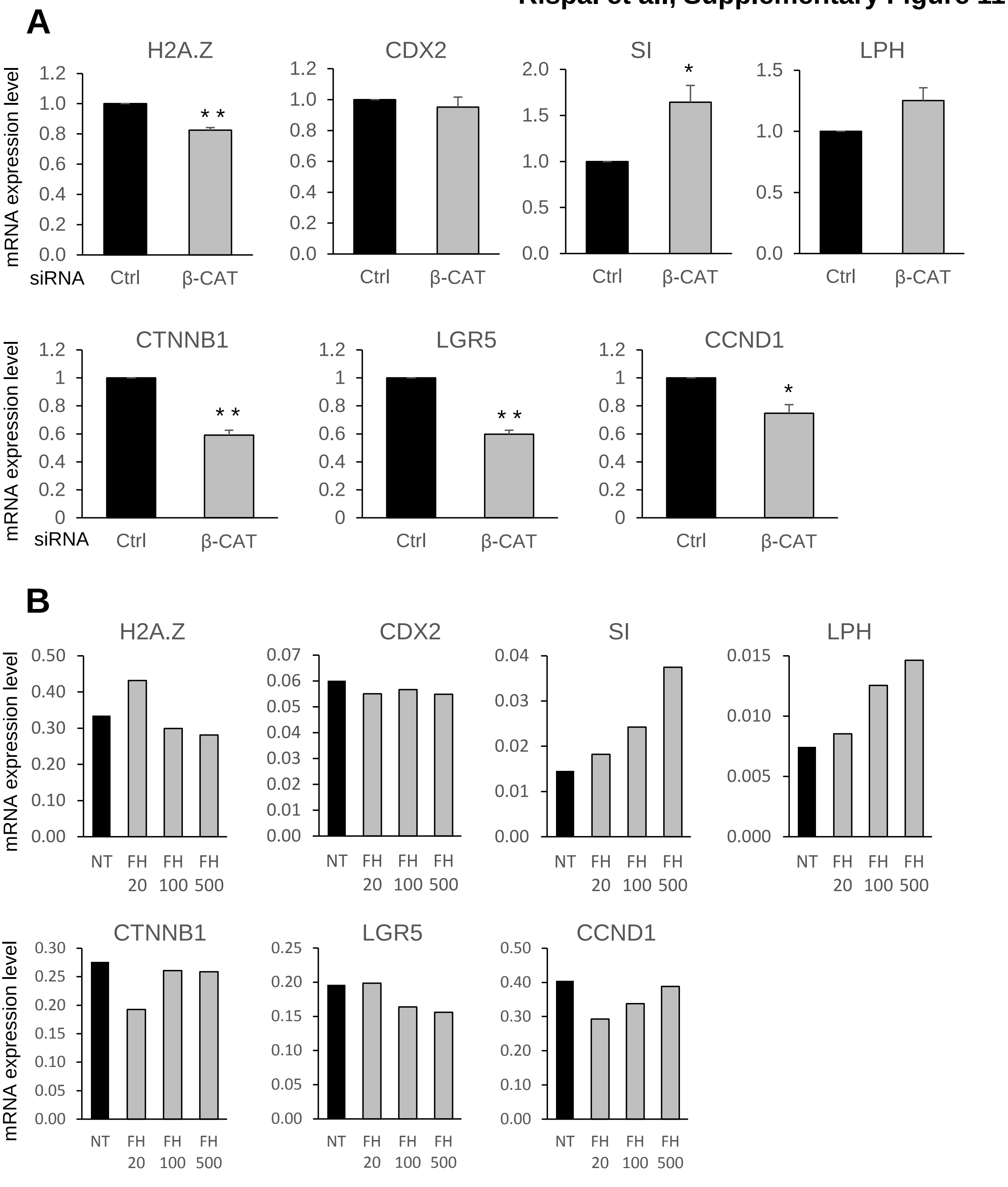

<u>Supplementary Figure 11</u> : Gene expression upon inhibition of the Wnt pathway activity.

A) Using another β-catenin -targetting siRNA, RT-qPCR analysis was done as in Figure 6, to control the silencing efficiency on β-catenin (*CTNNB1* gene expression), the expected impacts on Wnt targets (*LGR5* and *CCND1*) and to study the effects on indicated genes expression in Caco-2/15. The mean and standard error of 4 experiments is shown (**, $p<0.02$; *, $p<0.05$ vs control siRNA). B) Gene expression upon dose-response of FH535-mediated Wnt inhibition in Caco-2/15 cells. A representative experiment is shown.

A

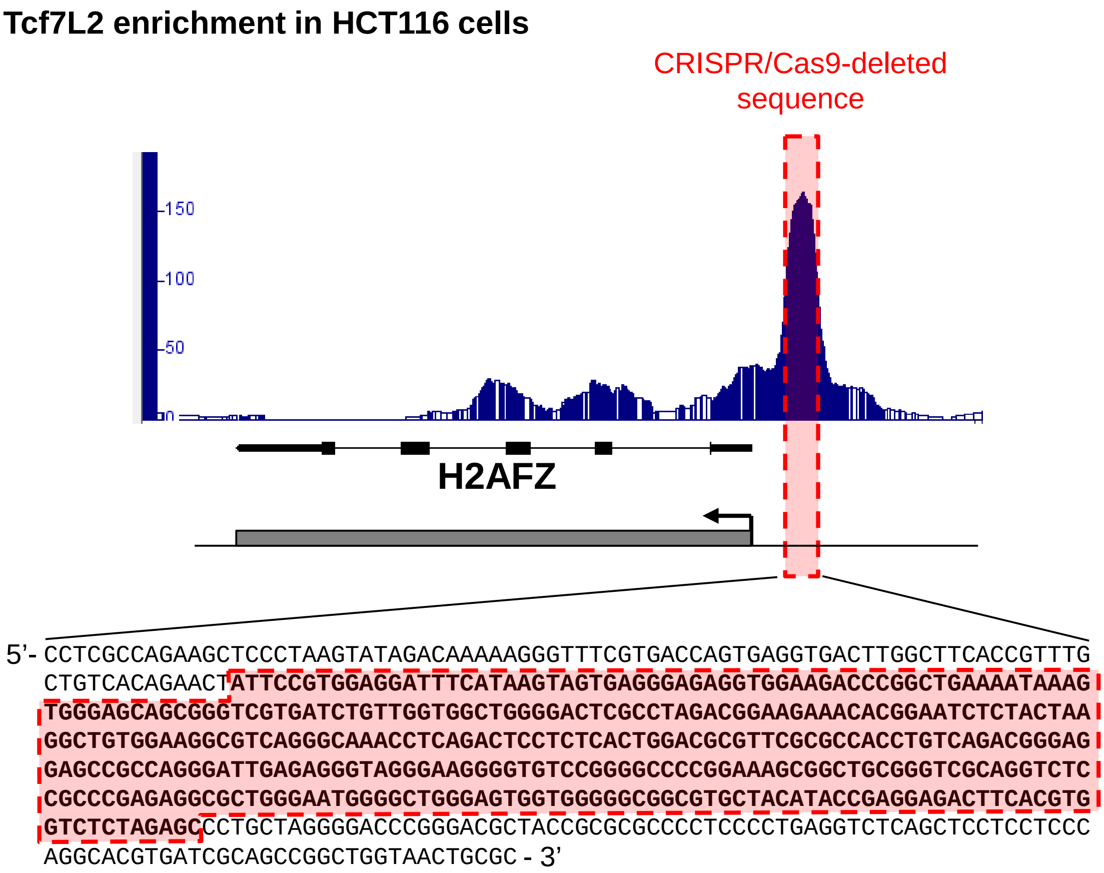

B

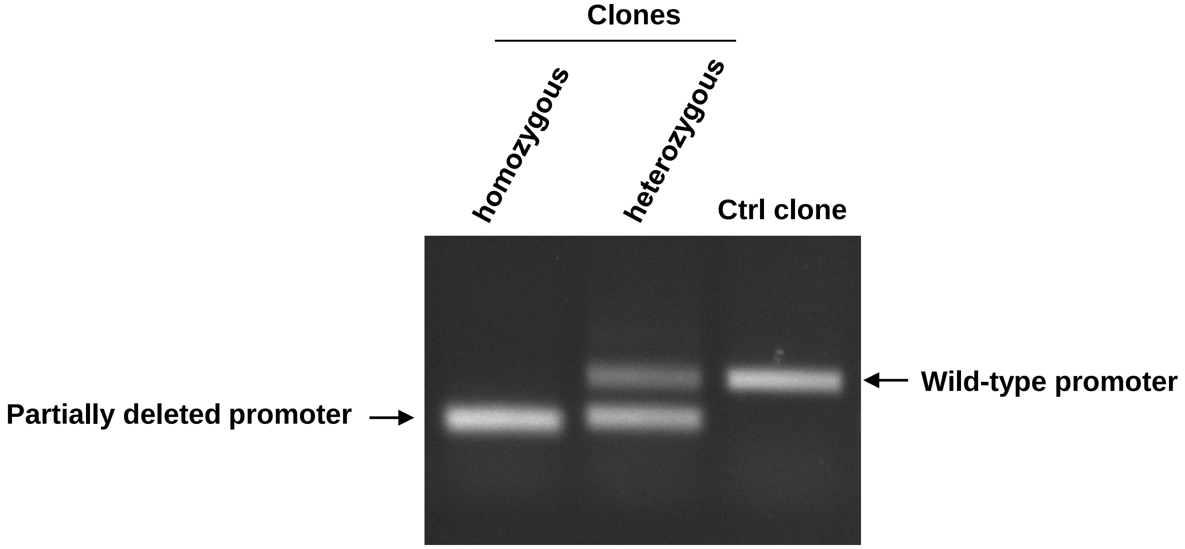

__Supplementary Figure 12__: CRISPR-Cas9 -mediated deletion of the TCF7L2-binding region on the H2A.Z promoter in HCT116 cells.

A) The strategy of is drawn. Data from Frietze et al., 2012 were analyzed using Integrated Genome Browser 9.0.2 to vizualize the peaks. B) Clones of CRISPR-edited HCT116 cells were isolated, grown, and genomic DNA were harvested and tested by PCR using primers described in Supplementary Table 6.

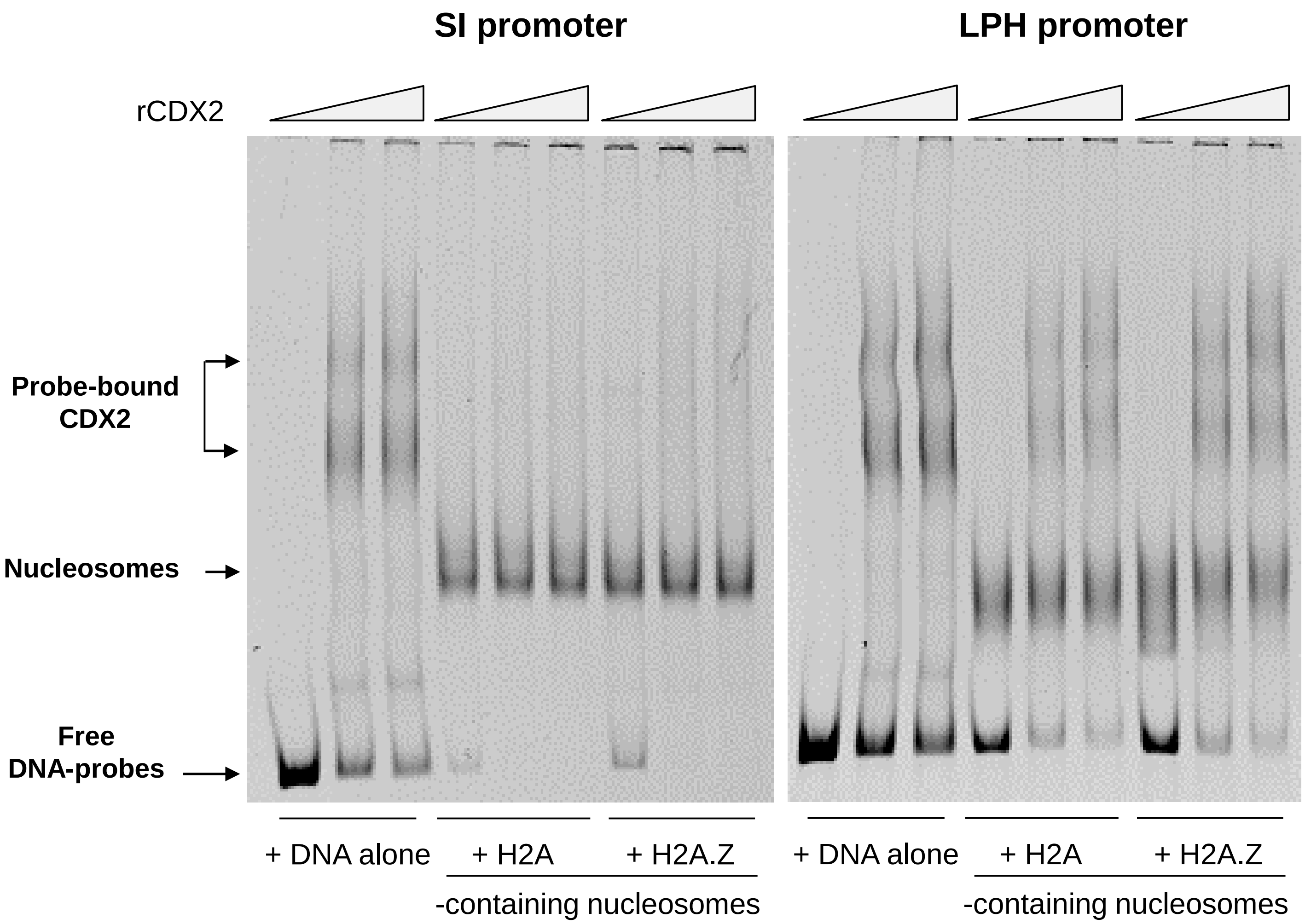

Supplementary Figure 13 : *In vitro* nucleosomes reconstitution and assays for CDX2 binding
Nucleosomes were reconstituted using recombinant H2A-H2B or H2A.Z-H2B, added to H3-H4 tetramers and flurorescence-labelled DNA probes for *SI* or *LPH* promoters, as described in the manuscript. Then, increasing quantities of recombinant CDX2 were added to such nucleosomes or to free DNA probes. Gels were analyzed using Typhoon device.

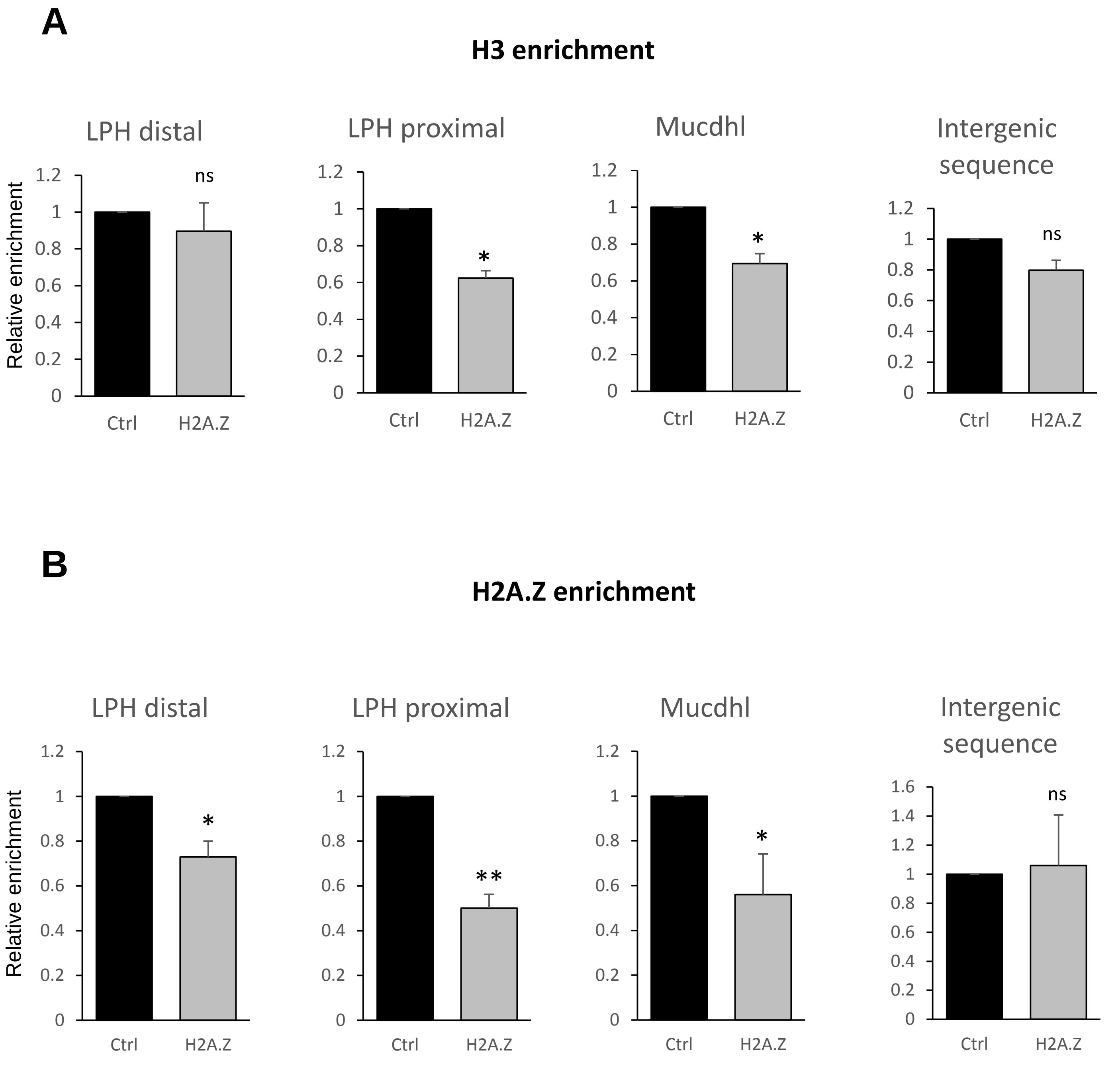

<u>Supplementary Figure 14</u> : ChIP analysis of H3 and H2A.Z binding on target genes promoters upon H2A.Z knock-down

A) Caco-2/15 cells were transfected using H2A.Z-targetting or control siRNA and enrichment in H3 binding on chromatin was analyzed, 72 hours later, by ChIP experiments, as described in the Methods section of the manuscript. Results are presented as the mean of percentage of input and standard error in three experiments.

B) Same as in A for H2A.Z ChIP on the same chromatin samples.

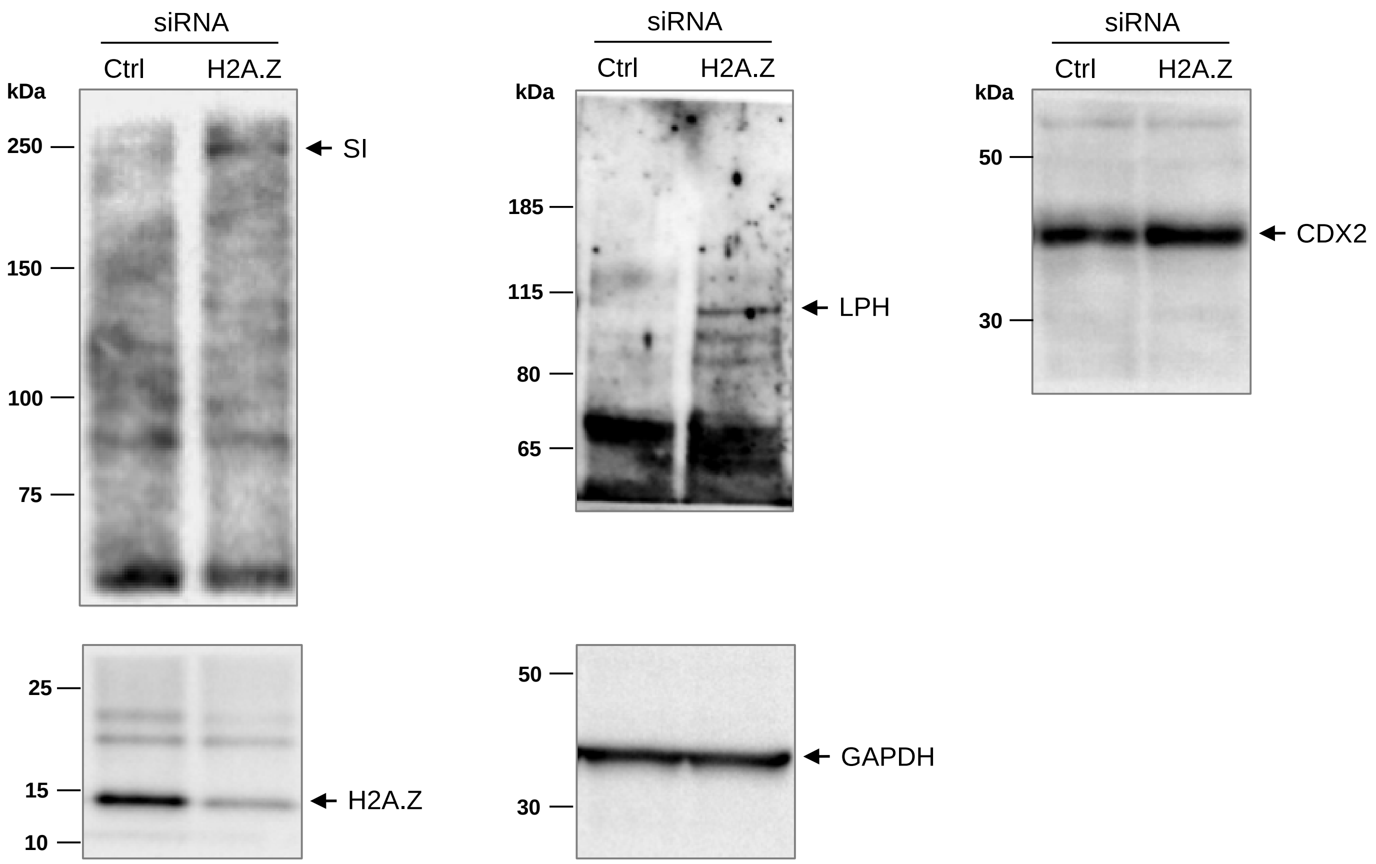

Supplementary Figure 15 : Original unprocessed western-blot panels from Figure 2A.

Genotyping primers

Gene	Forward primers	Reverse primers
**Tail samples**		
wild-type or floxed H2afz	CGCCTTGGTAATTCTATCTTCTCC	CGCCAGTTAACACACATGTGATC
wild-type Lgr5	ATACCCCATCCCTTTTGAGC	CTGCTCTCTGCTCCCAGTCT
Lgr5-CRE insert	GAACTTCAGGGTCAGCTTGC	CTGCTCTCTGCTCCCAGTCT
**Intestinal epithelium**		
wild-type and (native or recombined) floxed H2afz	CGCCTTGGTAATTCTATCTTCTCC	AAGCCTCCAACTTGCTCAAA

Supplementary Table 1 : Genotyping primer sequences.

siRNA sequence

siRNA	Sequence
Ctrl (1)	CAUGUCAUGUGUCACAUCU
Ctrl (2)	ACUCAAACUCACGAAGGAA
H2A.Z (1)	GUAGUGGGUUUUGAUUGAG
H2A.Z (2)	AAAGGACAACAGAAGACUG
CDX2	CCAGGACGAAAGACAAAUA
β-CATENIN (1)	GGAGCUAAAAUGGCAGUGC
β-CATENIN (2)	GUGGGUGGUAUAGAGGCUC

Supplementary Table 2 : Sequences of siRNAs.

Human primers

Gene	Forward primers	Reverse primers
β2M	AAAGATGAGTATGCCTGCCG	CCTCCATGATGCTGCTTACA
H2A.Z	CCTTTTCTCTGCCTTGCTTG	CGGTGAGGTACTCCAGGATG
CDX2	TCCCTCGGCAGCCAAGTGAA	TCGGCTTTCCTCCGGATGGT
SI	ATGTGAAGGTTGCCCAAAAC	AAAATTGGCCATGTTTTCCA
LPH	CCAGGAGATATGTTCAGTTC	CACTCTCTGTAAATTCTGGC
HNF1α	GCCCTGTACACCTGGTACGT	GCTCATCACCTGTGGGCTCT
MUCDHL L	CTCCCACCAACCAACCAC	CATATCCACCACCGAGAAGC
MUCDHL M	TGGAGGGAGAGGTTGTGCT	GGCCGCCACCTGTGGAGG
CTNNB1	TTCGTGCACATCAGGATACC	CTGCACAAACAATGGAATGG
LGR5	TGCTCTTCACCAACTGCATC	CTCAGGCTCACCAGATCCTC
CCND1	CGTGGCCTCTAAGATGAAGG	CTGGCATTTTGGAGAGGAAG
CDKN2A	CTGCCCAACGCACCGAATAG	ACCACCAGCGTGTCCAGGAA
HMGA1	ATGAGTGAGTCGAGCTCGAAG	TTAGGTGTTGGCACTTCGCTG
HMGA2	AAGCCACTGGAGAAAAACGGC	ATCCAACTGCTGCTGAGGTAG
KLF4	CCGCTCCATTACCAAGAGC	TGCCTTGAGATGGGAACTCT
ARHGEF2	ATCTACCCCTCCGACAGCTT	GGATTCCACGGATAGGGTTC
LDHA	TTGGTCCAGCGTAACGTGAAC	CCAGGATGTGTAGCCTTTGAG

Supplementary Table 3 : RT-qPCR human primer sequences.

Murine primers

Gene	Forward primers	Reverse primers
β2m	CCTGGTCTTTCTGGTGCTTG	TATGTTCGGCTTCCCATTCT
Si	GCTGGTCGATGGGGAGGA	CCAACGAGCACAGAGGTGGTAT
Lph	CCTTGAGCCCAAAGTGAAAG	GGACGTACAGCTCAGGAAGG
Chga	TTTTTGCCCTTCCTGTGAAC	GGTGTCGCAGGATAGAGAGG
Muc2	TGATGGCCATTGAGGTGGAG	CTGGCCCTTTGTGTTGTTGC
Muc4	GCTACAGAGCCAAGTGGACAC	AAGCCCATGAGCACCCGGTT
Lys	TGGCTGACTGGGTGTGTTTA	CACTGCAATTGATCCCACAG
Cryptdin	GCACAGAAGGCTCTGCTCTT	ACCCAGATTCCACATTCAGC
Cdx2	TCCCTAGGAAGCCAAGTGAA	CTGCGGTTCTGAAACCAAAT
Hnf1α	GCACACCCATGAAGACACAG	GGTTCCTACGCCCCTTCTTA
Gata4	CTGTGCCAACTGCCAGACTA	GCTTCCGTTTTCTGGTTTGA
Cdx1	TAAGACCCGAACCAAGGACA	GATCTTTACCTGCCGCTCTG
Hnf4α	GGTCAAGCTACGAGGACAGC	ATGTACTTGGCCCACTCGAC
Gata6	CAGCAAGCTGTTGTGGTC	GTCTGGTACATTTCCTCCG

Supplementary Table 4 : RT-qPCR murine primer sequences.

ChIP primers

Gene promoter	Forward primers	Reverse primers
LPH	AAAATTAGCCAGGCATCGTG	TTCAGACATTTTCCGGGTTC
MUCDHL	ACCCATGAGCCGCTCTCCAGTC	TCTGGCGTCTAGGACTGGCGCAGT
Intergenic	AGCACATGGGATTTTGCAGG	TTCCCTCCTTTGTGTCACCA
KLF4	ACTCGCCTTGCTGATTGTCT	CCCCAAAGTCAACGAAGAGA
H2AFZ (internal)	GCGTGCTACATACCGAGGAG	CGGGAGGGAAGAAACAGAG
H2AFZ (both sides)	ACTGATTGCATTGCATGGAC	CGGGAGGGAAGAAACAGAG
H2AFZ (downstream)	TGAGGTCTCAGCTCCTCCTC	CGGGAGGGAAGAAACAGAG

Supplementary Table 5 : Sequences of primers used in ChIP experiments.

CRISPR-Cas9 tools

Distal PAM :	5’-CACCG **GTCACAGAACTATTCCGTGG** -3’	
	5’-AAAC **CCACGGAATAGTTCTGTGAC** C-3’	
Proximal PAM :	5’-CACCG **CATACCGAGGAGACTTCACG** -3’	
	5’-AAAC **CGTGAAGTCTCCTCGGTATG** C-3’	
Screening primers	5’-ACTGATTGCATTGCATGGAC-3’	5’- CGGGAGGGAAGAAACAGAG -3’

Supplementary Table 6 : Tools used in HCT116 cells for CRISPR-Cas9 strategy to delete the TCF7L2 binding region on H2A.Z promoter.

Gene	Forward primers	Reverse primers
Si	Cy5-CTACTTTCAGGGTTAAGGCTTTT	TTGGATAAGGCTGCCAAAAT
Lph	Cy5-CGGCCCTGATCTTTACATTT	AGGAGGTATGTGGAACCCTTA

Supplementary Table 7 : Mouse genomic DNA amplification primer sequences.
