# Supplementary material for: The H2A.Z histone variant integrates Wnt signaling in intestinal epithelial homeostasis
Source: Nat Commun. 2019 Apr 23;10:1827. doi: 10.1038/s41467-019-09899-z (PMC6478875; doi:10.1038/s41467-019-09899-z)
Supplement: Supplementary file 3 — Reporting Summary [file 41467_2019_9899_MOESM3_ESM.pdf]

nature research

Corresponding author(s): Fabrice ESCAFFIT

Last updated by author(s): Mar 11, 2019

# Reporting Summary

Nature Research wishes to improve the reproducibility of the work that we publish. This form provides structure for consistency and transparency in reporting. For further information on Nature Research policies, see Authors & Referees and the Editorial Policy Checklist.

## Statistics

For all statistical analyses, confirm that the following items are present in the figure legend, table legend, main text, or Methods section.

| n/a | Confirmed | |
|---|---|---|
| ☐ | ☒ | The exact sample size (*n*) for each experimental group/condition, given as a discrete number and unit of measurement |
| ☐ | ☒ | A statement on whether measurements were taken from distinct samples or whether the same sample was measured repeatedly |
| ☐ | ☒ | The statistical test(s) used AND whether they are one- or two-sided<br>*Only common tests should be described solely by name; describe more complex techniques in the Methods section.* |
| ☒ | ☐ | A description of all covariates tested |
| ☒ | ☐ | A description of any assumptions or corrections, such as tests of normality and adjustment for multiple comparisons |
| ☒ | ☐ | A full description of the statistical parameters including central tendency (e.g. means) or other basic estimates (e.g. regression coefficient) AND variation (e.g. standard deviation) or associated estimates of uncertainty (e.g. confidence intervals) |
| ☒ | ☐ | For null hypothesis testing, the test statistic (e.g. *F*, *t*, *r*) with confidence intervals, effect sizes, degrees of freedom and *P* value noted<br>*Give P values as exact values whenever suitable.* |
| ☒ | ☐ | For Bayesian analysis, information on the choice of priors and Markov chain Monte Carlo settings |
| ☒ | ☐ | For hierarchical and complex designs, identification of the appropriate level for tests and full reporting of outcomes |
| ☒ | ☐ | Estimates of effect sizes (e.g. Cohen's *d*, Pearson's *r*), indicating how they were calculated |

*Our web collection on statistics for biologists contains articles on many of the points above.*

## Software and code

Policy information about availability of computer code

| | |
|---|---|
| Data collection | Bio-Rad CFX Manager version 3.1<br>Image Lab Touch version 1.2.0.12<br>MetaMorph version 7.1.0.0<br>E-Capt version 15.06<br>CellQuest Pro version 6.1 |
| Data analysis | Microsoft Office Professionel Plus 2016<br>GraphPad Prism 5<br>Bio-rad Image Lab version 5.2.1<br>Image J 1.51h<br>IGB 9.0.0<br>Flow-Jo v10 |

For manuscripts utilizing custom algorithms or software that are central to the research but not yet described in published literature, software must be made available to editors/reviewers. We strongly encourage code deposition in a community repository (e.g. GitHub). See the Nature Research guidelines for submitting code & software for further information.

## Data

Policy information about availability of data

All manuscripts must include a data availability statement. This statement should provide the following information, where applicable:

- Accession codes, unique identifiers, or web links for publicly available datasets
- A list of figures that have associated raw data
- A description of any restrictions on data availability

The authors declare that all data supporting the findings of this study are available within the paper and its supplementary information files or from the

corresponding author [FE] upon request.

# Field-specific reporting

Please select the one below that is the best fit for your research. If you are not sure, read the appropriate sections before making your selection.

☒ Life sciences ☐ Behavioural & social sciences ☐ Ecological, evolutionary & environmental sciences

For a reference copy of the document with all sections, see nature.com/documents/nr-reporting-summary-flat.pdf

# Life sciences study design

All studies must disclose on these points even when the disclosure is negative.

| | |
|---|---|
| Sample size | Experiments using cell cultures were systematically done more than three times in order to ensure reproducibility.<br>Animals cohorts were determine based on preliminary experiments evaluating biological effects amplitudes. |
| Data exclusions | No data were excluded from analysis. |
| Replication | Reproducibility was evaluated by comparing means and deviation of experimental replicates and significativity of effects were calculated using statistical tests indicated in the manuscript. |
| Randomization | All samples and animals of this study was analyzed independently and randomly. |
| Blinding | Samples from animals were collected and analyzed in a blinded manner. |

# Reporting for specific materials, systems and methods

We require information from authors about some types of materials, experimental systems and methods used in many studies. Here, indicate whether each material, system or method listed is relevant to your study. If you are not sure if a list item applies to your research, read the appropriate section before selecting a response.

## Materials & experimental systems

| n/a | Involved in the study | |
|---|---|---|
| ☐ | ☒ | Antibodies |
| ☐ | ☒ | Eukaryotic cell lines |
| ☒ | ☐ | Palaeontology |
| ☐ | ☒ | Animals and other organisms |
| ☒ | ☐ | Human research participants |
| ☒ | ☐ | Clinical data |

## Methods

| n/a | Involved in the study | |
|---|---|---|
| ☒ | ☐ | ChIP-seq |
| ☐ | ☒ | Flow cytometry |
| ☒ | ☐ | MRI-based neuroimaging |

## Antibodies

| | |
|---|---|
| Antibodies used | H2A.Z (abcam, ab4174, Polyclonal, GR3198864-1), SI (Santa-Cruz, sc-393470, C-8 J3013), Lysosyme (abcam, ab108508, EPR2994(2), GR109789-10), Ki67 (abcam, ab15580, Polyclonal, GR85841-1), SI (Novus, NBP1-62362, Polyclonal), LPH (Biorbyt, orb184881, Polyclonal, BR4596), CDX2 (abcam, ab88129, Polyclonal, GR257577-2), PARP (Cell Signaling, 9542, Polyclonal, 14), CDKN2A (abcam, ab108349, EPR1473), GAPDH (Chemicon, Mab374, 6C5, 2955484), CDX2 (Bethyl, A300-691A, Polyclonal), TCF7L2 (Cell Signaling, 2569, C48H11, 4), H3 (abcam, ab1791, Polyclonal, GR3181935-1). |
| Validation | H2A.Z: validated for WB and ChIP experiments in human by the manufacturer and us (see decrease after siRNA directed against H2A.Z)<br>SI: validated for IHC experiment in mouse by the manufacturer and us (staining located in intestinal villus as expected)<br>Lysosyme: validated for IHC experiment in mouse by the manufacturer and us (staining located in intestinal crypts as expected)<br>Ki67: validated for IHC experiment in mouse by the manufacturer and us (staining located in intestinal crypts as expected)<br>SI: validated for WB in human by the manufacturer and us (band located at expected molecular weight)<br>LPH: validated for WB in human by the manufacturer and us (band located at expected molecular weight)<br>CDX2: validated for WB in human by the manufacturer and us (band located at expected molecular weight)<br>PARP: validated for WB in human by the manufacturer and us (band located at expected molecular weight)<br>CDKN2A: validated for WB in human by the manufacturer and us (band located at expected molecular weight)<br>GAPDH: validated for WB in human by the manufacturer and us (band located at expected molecular weight)<br>CDX2: validated for ChIP experiment in human in the literature (Verzi, M. P., Shin, H., Ho, L.-L., Liu, X. S. & Shivdasani, R. A. Essential and redundant functions of caudal family proteins in activating adult intestinal genes. Mol. Cell. Biol. 31, 2026–2039 (2011))<br>TCF7L2: validated for ChIP experiment in human by the manufacturer and in the literature (Frietze, S. et al. Cell type-specific |

binding patterns reveal that TCF7L2 can be tethered to the genome by association with GATA3. Genome Biol. 13, R52 (2012))
H3: validated for ChIP experiment in human by the manufacturer

# Eukaryotic cell lines

Policy information about cell lines

| | |
|---|---|
| Cell line source(s) | HIEC and Caco-2/15 cell lines were provided by the Pr Jean-François Beaulieu's lab (Université de Sherbrooke, Quebec, Canada)<br>HCT116 cell line was a gift from Dr Patrick Calsou (IPBS, Toulouse, France) |
| Authentication | The names of the used cell lines are authentic and previously published |
| Mycoplasma contamination | All cell lines were regularly tested for absence of mycoplasma contamination by using the MycoAlert Mycoplasma Detection Kit (Lonza #LT07-703) |
| Commonly misidentified lines<br>(See ICLAC register) | N/A |

# Animals and other organisms

Policy information about studies involving animals; ARRIVE guidelines recommended for reporting animal research

| | |
|---|---|
| Laboratory animals | Animals used in this study are in C57Bl/6J genetic background and breaded to generate Lgr5-CreERT2/H2afzfl/fl mouse strain. All along this study, only 6-8 week-old male mice were used. |
| Wild animals | This study did not involve wild animals. |
| Field-collected samples | This study did not involve samples collected from the field. |
| Ethics oversight | This study was approved by the CBI Ethics Committee of the institute “Centre de Biologie Intégrative” (FR3743) and was authorized by the French Ministry of Education and Research (approval APAFIS #4528-2016031109479615 v3) |

Note that full information on the approval of the study protocol must also be provided in the manuscript.

# Flow Cytometry

## Plots

Confirm that:

☒ The axis labels state the marker and fluorochrome used (e.g. CD4-FITC).

☒ The axis scales are clearly visible. Include numbers along axes only for bottom left plot of group (a 'group' is an analysis of identical markers).

☒ All plots are contour plots with outliers or pseudocolor plots.

☒ A numerical value for number of cells or percentage (with statistics) is provided.

## Methodology

| | |
|---|---|
| Sample preparation | Caco-2/15 cells were transfected with siRNA directed against H2A.Z. Then the cells were treated with EdU, fixed and incubated with PI (Propidium Iodide). Finally, EdU and PI stainings were measured using FACSCalibur. |
| Instrument | BD FACS Calibur |
| Software | CellQuest Pro version 6.1 for the acquisition<br>Flow-Jo v10 for the analysis |
| Cell population abundance | Measured cell populations were out of at least 10.000 events, after gating, per condition. |
| Gating strategy | - In the SSC/FSC graph we maked a gate to select the cells and exclude the debris.<br>- In the Area/Height graph we maked a gate to select single cells (cells with the area proportionnal to the height) and exclude doublets.<br>- In the EdU/PI graph we distinguished the cell cycle phases : G0/G1 (low EdU, low PI), S (High EdU) and G2 (low EdU, High PI) |

☒ Tick this box to confirm that a figure exemplifying the gating strategy is provided in the Supplementary Information.
